# Off-pump bilateral internal thoracic artery grafting in patients with left main coronary artery disease

**DOI:** 10.1186/s13019-024-02582-5

**Published:** 2024-02-09

**Authors:** Kohei Hachiro, Noriyuki Takashima, Tomoaki Suzuki

**Affiliations:** Division of Cardiovascular Surgery, Department of Surgery, Shiga University of Medical Science, Setatsukinowa-cho, Otsu, 520-2192 Shiga Japan

**Keywords:** Coronary artery bypass grafting, Left main coronary artery disease, Bilateral internal thoracic artery

## Abstract

**Background:**

To compare postoperative outcomes in patients with left main coronary artery disease who underwent off-pump isolated coronary artery bypass grafting for multivessel disease using either skeletonized bilateral or single internal thoracic artery (ITA).

**Methods:**

Among 1583 patients who underwent isolated coronary artery bypass grafting (CABG) in our hospital between 2002 and 2022, 604 patients with left main coronary artery disease underwent single (*n* = 169) or bilateral (*n* = 435) ITA grafting. We compared postoperative outcomes between the two groups after adjusting preoperative characteristics using inverse probability of treatment weighting.

**Results:**

After adjustment using inverse probability of treatment weighting method, the sum of weights was 599.74 in BITA group and 621.64 in SITA group. There was no significant difference in postoperative deep sternal wound infection (*p* = 0.227) and 30-day mortality (*p* = 0.612). Follow-up was completed in 98.7% (596/604) of the patients, and the mean follow-up duration was 6.7 years. At 10 years, the overall survival following bilateral versus single ITA grafting was 71.2% and 60.6%, respectively (log-rank test, *p* = 0.040), and freedom from major adverse cardiac and cerebrovascular events (MACCE) was 63.3% and 46.3%, respectively (log-rank test, *p* = 0.008). In multivariate Cox proportional hazard models, bilateral ITA grafting was significantly associated with a lower risk of all-cause death (hazard ratio [HR]: 0.706, 95% confidence interval [CI]: 0.504–0.987; *p* = 0.042) and MACCE (HR: 0.671, 95% CI: 0.499–0.902; *p* = 0.008).

**Conclusions:**

Bilateral skeletonized ITA grafting is associated with lower rates of all-cause death and MACCE than single ITA grafting in patients with left main coronary artery disease undergoing off-pump CABG.

**Supplementary Information:**

The online version contains supplementary material available at 10.1186/s13019-024-02582-5.

## Introduction

With improvements in coronary intervention and advances in medical treatment, several randomized trials have shown that percutaneous coronary intervention (PCI) with drug-eluting stents is an alternative for selected patients with left main coronary artery disease (LMCAD) [1–3]. However, as stated in recent guidelines for coronary artery revascularization [4, 5], coronary artery bypass grafting (CABG) remains an important treatment for LMCAD, especially in patients with complex coronary artery disease.

Bilateral internal thoracic artery (BITA) grafting has been reported to be associated with better long-term outcomes than single internal thoracic artery (SITA) grafting [6–9]. However, little is known about BITA grafting for patients with LMCAD. The aim of this study was to evaluate the efficacy of BITA grafting in patients with LMCAD who underwent off-pump isolated CABG.

### Patients and methods

All patients previously provided informed consent to use their medical records for research purposes, and the ethics committee of Shiga University of Medical Science approved this study (Reg. No. R2022-040; approval date: June 17, 2022).

Between January 2002 and December 2022, 1,583 patients underwent isolated CABG at our institution (Figure E1). Among them, 641 patients had LMCAD. We excluded 8 patients who underwent grafting without using the ITA, 10 patients whose coronary anatomy presented only one target vessel in the left coronary artery system, 4 patients who preoperatively had emergent percutaneous cardiopulmonary bypass support at another hospital during cardiopulmonary resuscitation for cardiac arrest caused by acute myocardial infarction, 10 patients who underwent grafting under cardiopulmonary bypass support because of unstable hemodynamic condition, and 5 patients who had undergone cardiac surgery before coronary artery bypass grafting. Finally, 604 patients underwent CABG surgery using SITA (*n* = 169) or BITA (*n* = 435) (Figure E2).

### Outcome measures and definitions

The primary outcome measure was all-cause death. The secondary outcome was the incidence of major adverse cardiac and cerebrovascular events (MACCE), defined as a composite of all-cause death, non-fatal myocardial infarction, non-fatal heart failure, non-fatal stroke and need for repeat revascularization. Non-fatal MI, non-fatal heart failure, and non-fatal stroke were defined as new admissions with a diagnosis of these diseases during the follow-up period that did not result in death. LMCAD was defined as a stenosis of greater than 50% according to visual assessment of the preoperative coronary angiogram. Postoperative outcomes, including various postoperative complications such as stroke and deep sternal wound infection, were defined based on the Japan Cardiovascular Surgery Database protocols, which are almost identical to those used in The Society of Thoracic Surgeons National Database.

Patients come to our outpatient follow-up once a year after CABG. Data for all perioperative variables were obtained from the database or directly from individual electronic medical records at our hospital. Information on late follow-up was collected from hospital records or primary care doctors. In case the information was still inadequate, we sought further details from relatives by telephone.

### Surgical treatment and graft arrangement

We used the off-pump technique for CABG whenever hemodynamically possible. In our cohort of the present study, the off-pump technique was used in all included patients. Details of surgical techniques, including graft harvest and graft arrangement, have been published previously [10]. The left anterior descending (LAD) artery was always revascularized using in situ grafting of the ITA at first. A second ITA and/or saphenous vein, as either a sequential or individual graft, was grafted to the circumflex and/or diagonal branches. The inferior wall was revascularized using the saphenous vein, in situ gastroepiploic artery (GEA), or both. Use of the GEA required stenosis of > 90% in the target vessels. In most patients who underwent BITA grafting, the in situ right ITA was tunneled through a right-sided pericardial incision and routed anterior to the aorta across the midline for grafting to the LAD, and the in situ left ITA was used for the circumflex branches, diagonal branches, or both. Radial artery grafts and endoscopic vein harvest were not used in any of these cases.

When the ITA was injured at its proximal portion, or when the right ITA was too short for grafting to the LAD artery, we constructed a composite graft or anastomosed its proximal portion to the ascending aorta. The free ITA was anastomosed to the other ITA or ascending aorta in an end-to-side manner in BITA group, whereas in SITA group the free ITA was anastomosed to the saphenous vein graft or ascending aorta in an end-to-side manner. We routinely performed computed tomographic scans and epiaortic ultrasound to assess the severity and location of ascending aortic atherosclerosis to prevent complications related to manipulating the ascending aorta. When the surgeon judged that partial clamping of the ascending aorta carried a risk of embolism, a proximal anastomotic device (Novare Enclose; Novare Surgical Systems, Cupertino, CA, USA) was used.

BITA grafting was preferred for revascularization of the left coronary territory whenever anatomically possible, even if the patient had poor blood sugar control before surgery, and in emergency operations. We measured blood pressure non-invasively in both upper arms preoperatively. The measurement was performed at rest in the supine position in both upper arms simultaneously. When there was a difference of ≥ 20 mmHg between blood pressure measurements, we did not use the ITA on the side with the lower pressure. All CABG procedures were mainly performed by two high-volume surgeons who were familiar with the technique (Figure E3).

### Statistical analysis

Continuous variables are presented as mean ± standard deviation, or median and interquartile range, whereas categorical variables are presented as percentages. Comparisons of clinical characteristics between the 2 groups were performed using the unpaired t-test for normally distributed variables, the Mann–Whitney U test for skewed variables, and Pearson’s χ2 test for categorical variables. Probabilities of survival were estimated using the Kaplan–Meier method, in which patients still alive were censored at the date of their last follow-up; the log-rank test was used for comparisons. Univariate and multivariate logistic regression analyses were performed to identify independent predictors of 30-day mortality. Univariate and multivariate Cox proportional hazards regression analyses were performed to analyze the all-cause death and MACCE. Variables reaching a *P* value of < 0.050 in the univariate analysis or those that were considered clinically important were entered into the multivariate model. All statistical testing was 2-sided, and results were considered statistically significant at *P* < 0.050.

We adjusted patients’ baseline characteristics using weighted logistic regression analysis and inverse probability of treatment weighting (IPTW) to reduce any effect of selection bias and potential confounding factors. Weights for patients receiving BITA grafting were the inverse of propensity scores, and weights for patients receiving SITA grafting were the inverse of 1 – the propensity score. We used the following 17 adjustment variables to derive the propensity score: age, sex, body mass index, hypertension, diabetes mellitus (DM), dyslipidemia, smoking history, previous cerebrovascular accident, history of PCI, peripheral artery disease, three-vessel disease, hemoglobin A1c, estimated glomerular filtration rate (eGFR) < 30 ml/min/1.73m2, emergency operation, acute myocardial infarction, left ventricular ejection fraction < 50% and intra-aortic balloon pumping. The model was well calibrated (Hosmer–Lemeshow test, *P* = 0.387), with reasonable discrimination (C-statistic, 0.748). Absolute standardized mean differences were calculated to compare the balance in baseline characteristics between the BITA and SITA groups in the unweighted cohort and the weighted cohort. An absolute standardized mean difference of > 0.100 was considered a meaningful imbalance [11]. All statistical analyses were performed using SPSS, version 25.0 (IBM Corp., Armonk, NY, USA) and SAS, version 9.4 (SAS Institute, Cary, NC, USA).

## Results

The blood pressure in left upper arm was more than 20mmHg lower than right upper arm preoperatively in 10 patients, so they underwent grafting using right ITAs. We injured and could not use left ITAs in 2 patients, so they went grafting using right ITAs. Right ITAs were too short to revascularize the LAD artery in 12 patients in the BITA group. Of them, the proximal portion of right ITAs were cut and anastomosed to left ITAs in 9 patients, to saphenous vein graft (SVG) in 2 patients, and to ascending aorta in 1 patient. LITAs’ pulsations were weak in 3 patients in whom we could not measure blood pressure in both upper arms preoperatively in emergency operation. We cut the proximal portion of LITAs and anastomosed the proximal portion to ascending aorta in 1 patient, and to SVG in 2 patients.

The mean age of our study population was 69.3 ± 10.0 years, and 482 (79.8%) were males. After adjustment using IPTW, the sum of weights was 599.74 in BITA group and 621.64 in SITA group. Their preoperative characteristics in the 2 groups were well balanced After IPTW adjustment (Table 1).

**Table 1:** Preoperative patient characteristics

	Unweighted				Weighted			
	BITA	SITA			BITA	SITA		
	(*n* = 435)	(*n* = 169)	*P* value	ASMD	(SoW = 599.74)	(SoW = 621.64)	*P* value	ASMD
Age (year)	67.6 ± 9.7	73.6 ± 9.3	< 0.001	0.631	69.1 ± 9.7	68.9 ± 10.4	0.675	0.020
Sex (male)	361 (83.0%)	121 (71.6%)	0.004	0.275	482.90 (80.5%)	505.88 (81.4%)	0.702	0.023
Body mass index (kg/m^2^)	23.8 ± 3.0	23.1 ± 3.7	0.028	0.208	23.7 ± 3.1	23.8 ± 3.5	0.465	0.030
Hypertension	302 (69.4%)	130 (76.9%)	0.057	0.170	430.34 (71.8%)	435.13 (70.0%)	0.500	0.040
Diabetes mellitus	230 (52.9%)	93 (55.0%)	0.634	0.042	324.49 (54.1%)	357.08 (57.4%)	0.191	0.066
Dyslipidemia	242 (55.6%)	86 (50.9%)	0.294	0.094	324.49 (54.1%)	338.99 (54.5%)	0.881	0.008
Smoking history	274 (63.0%)	84 (49.7%)	0.003	0.271	363.77 (60.7%)	378.78 (60.9%)	0.921	0.004
Previous CVD	48 (11.0%)	23 (13.6%)	0.379	0.079	70.42 (11.7%)	75.02 (12.1%)	0.860	0.012
Previous PCI	134 (30.8%)	39 (23.1%)	0.050	0.174	174.30 (29.1%)	156.90 (25.2%)	0.134	0.088
PAD	23 (5.3%)	12 (7.1%)	0.393	0.075	37.56 (6.3%)	37.19 (6.0%)	0.838	0.012
Three-vessel disease	325 (74.7%)	135 (79.9%)	0.167	0.124	456.98 (76.2%)	461.90 (74.3%)	0.444	0.044
Hemoglobin A1c (%)	6.4 ± 1.2	6.3 ± 1.0	0.380	0.091	6.4 ± 1.1	6.4 ± 1.0	0.797	0.019
eGFR < 30 ml/min/1.73m^2^	57 (13.1%)	41 (24.3%)	0.003	0.290	99.32 (16.6%)	108.42 (17.4%)	0.683	0.021
Emergency operation	129 (29.7%)	91 (53.8%)	< 0.001	0.504	213.18 (35.5%)	199.87 (32.2%)	0.210	0.070
Acute MI	128 (29.4%)	67 (39.6%)	0.020	0.216	194.54 (32.4%)	178.98 (28.8%)	0.167	0.078
LVEF < 50%	135 (31.0%)	76 (45.0%)	0.002	0.291	210.05 (35.0%)	212.25 (34.1%)	0.747	0.019
Preoperative IABP	57 (13.1%)	54 (32.0%)	< 0.001	0.464	108.14 (18.0%)	107.53 (17.3%)	0.737	0.018
STS score (%)	1.33 (0.72–2.68)	3.42 (1.70–9.12)	< 0.001	0.716	1.60 (0.84–3.39)	1.84 (0.81–4.01)	0.090	0.098
EuroSCORE II (%)	1.70 (1.05–3.43)	4.18 (1.95–14.00)	< 0.001	0.630	2.07 (1.14–5.18)	2.12 (1.04–5.25)	0.532	0.036

ASMD: absolute standardized mean difference; BITA: bilateral internal thoracic artery; CVD: cerebrovascular disease; eGFR: estimated glomerular filtration rate; EuroSCORE: European System for Cardiac Operative Risk Evaluation; IABP: intra-aortic balloon pumping; LVEF: left ventricular ejection fraction; MI: myocardial infarction; PAD: peripheral artery disease; PCI: percutaneous coronary intervention; SITA: single internal thoracic artery; SoW: sum of weights; STS: Society of Thoracic Surgeons

### Early outcomes

Operative and postoperative outcomes are shown in Table 2. The BITA group had longer operation times than did the SITA group (245 ± 60 min vs. 230 ± 58 min, respectively; *p* < 0.001). No significant difference in the number of distal anastomoses was found between the two groups (*p* = 0.276), but the BITA group had a greater number of grafts than did the SITA group (2.7 ± 0.5 vs. 2.2 ± 0.4, respectively; *p* < 0.001) and higher rates of GEA use (51.7% vs. 35.6%, respectively; *p* < 0.001), as well as lower rates of proximal anastomosis to the ascending aorta (20.3% vs. 84.5%, respectively; *p* < 0.001), sequential grafting (44.6% vs. 70.4%, respectively; *p* < 0.001), and saphenous vein graft use (21.5% vs. 87.8%, respectively; *p* < 0.001). There was a higher rate of intensive care unit stay > 48 h (16.9% vs. 11.9%, respectively; *p* = 0.012) and ventilation time > 48 h (9.8% vs. 6.0%, respectively; *p* = 0.013) in the SITA group than in the BITA group. There was no significant difference in postoperative deep sternal wound infection (*p* = 0.227) and 30-day mortality (*p* = 0.612). Multivariate logistic regression analyses showed that eGFR < 30 ml/min/1.73m2 was the only predictor of 30-day mortality (odds ratio: 22.856, 95% confidence interval (CI): 5.538–94.327; *P* < 0.001) (Table E1).

**Table 2:** Operative and postoperative data

	Unweighted			Weighted		
	BITA	SITA		BITA	SITA	
	(*n* = 435)	(*n* = 169)	*P* value	(SoW = 599.74)	(SoW = 621.64)	*P* value
Operative data						
Operation time (m)	246 ± 60	225 ± 59	< 0.001	245 ± 60	230 ± 58	< 0.001
Proximal anastomosis to aorta	83 (19.1%)	144 (85.2%)	< 0.001	121.52 (20.3%)	525.43 (84.5%)	< 0.001
Partial clamp	70 (16.1%)	109 (64.9%)	< 0.001	101.64 (16.9%)	389.96 (62.7%)	< 0.001
Anastomotic device	13 (3.0%)	34 (20.2%)	< 0.001	19.88 (3.3%)	135.47 (21.8%)	< 0.001
No. of distal anastomoses	3.3 ± 1.0	3.3 ± 0.9	0.635	3.3 ± 1.0	3.3 ± 1.0	0.276
No. of grafts	2.7 ± 0.5	2.2 ± 0.4	< 0.001	2.7 ± 0.5	2.2 ± 0.4	< 0.001
Sequential grafting	199 (45.7%)	129 (76.3%)	< 0.001	267.46 (44.6%)	437.90 (70.4%)	< 0.001
GEA use	229 (62.6%)	58 (34.3%)	< 0.001	310.21 (51.7%)	221.35 (35.6%)	< 0.001
SVG use	86 (19.8%)	151 (89.3%)	< 0.001	129.01 (21.5%)	545.53 (87.8%)	< 0.001
Postoperative data						
Myocardial infarction	3 (0.7%)	2 (1.2%)	0.548	3.55 (0.6%)	6.50 (1.0%)	0.381
DSWI	7 (1.6%)	1 (0.6%)	0.229	8.68 (1.4%)	4.52 (0.7%)	0.227
Stroke	4 (0.9%)	2 (1.2%)	0.770	7.89 (1.3%)	3.07 (0.5%)	0.131
Intraoperative IABP	7 (1.6%)	4 (2.4%)	0.533	10.70 (1.78%)	20.78 (3.34%)	0.084
Intraoperative PCPS	0 (0%)	2 (1.2%)	0.158	0 (0%)	7.05 (1.1%)	0.008
ICU stay > 48 h	38 (8.7%)	43 (25.4%)	< 0.001	71.19 (11.9%)	104.99 (16.9%)	0.012
Ventilation > 48 h	17 (3.9%)	27 (16.0)	< 0.001	35.90 (6.0%)	60.99 (9.8%)	0.013
30-day mortality	5 (1.1%)	5 (3.0%)	0.199	8.39 (1.4%)	6.70 (1.1%)	0.612

BITA: bilateral internal thoracic arteries; DSWI: deep sternal wound infection; GEA: gastroepiploic artery; ICU: intensive care unit; LAD: left anterior descending artery; PCPS: percutaneous cardiopulmonary support; SITA: single internal thoracic artery; SoW: sum of weights; SVG: saphenous vein graft

### Long-term outcomes

**Fig. 1:**
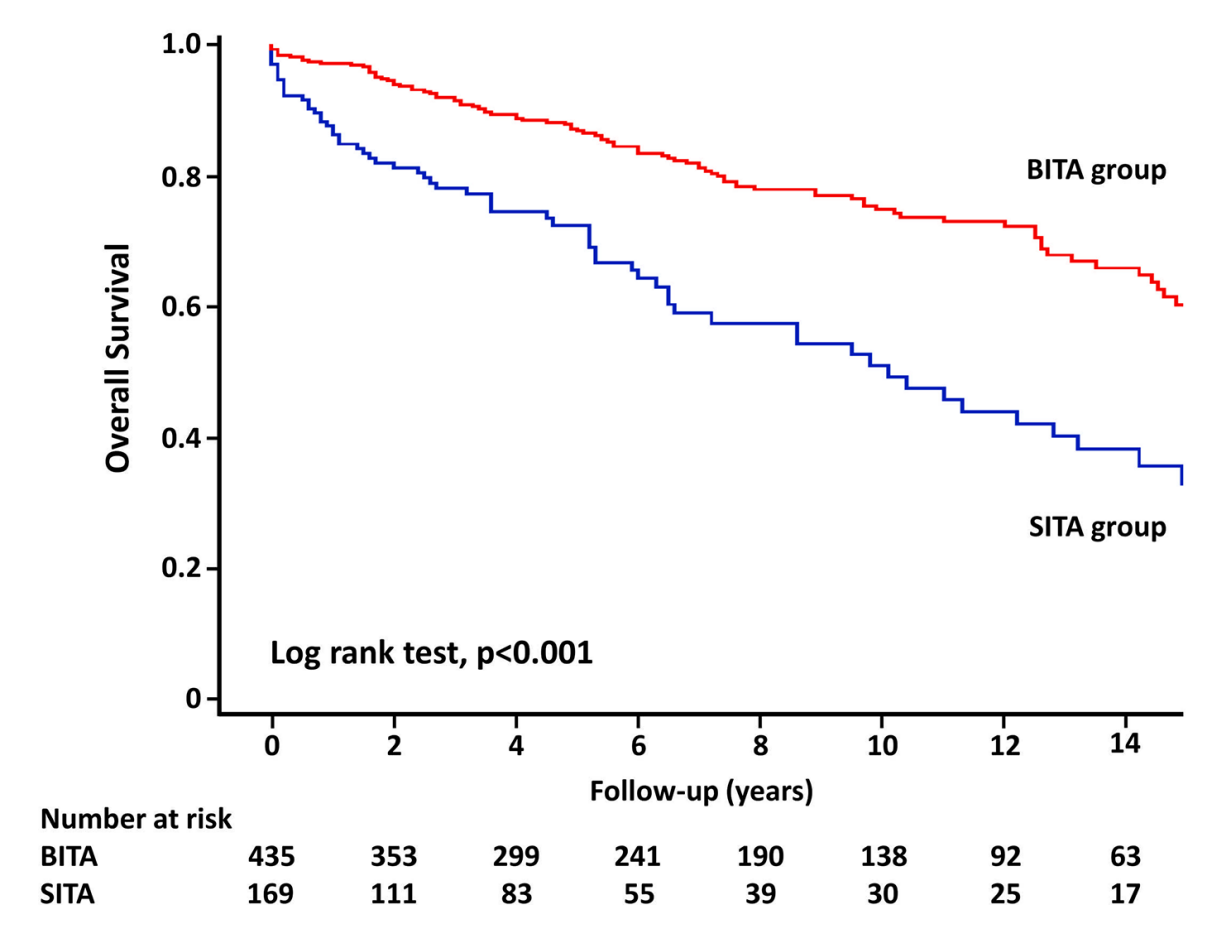
Overall survival in unweighted cohort

**Fig. 2:**
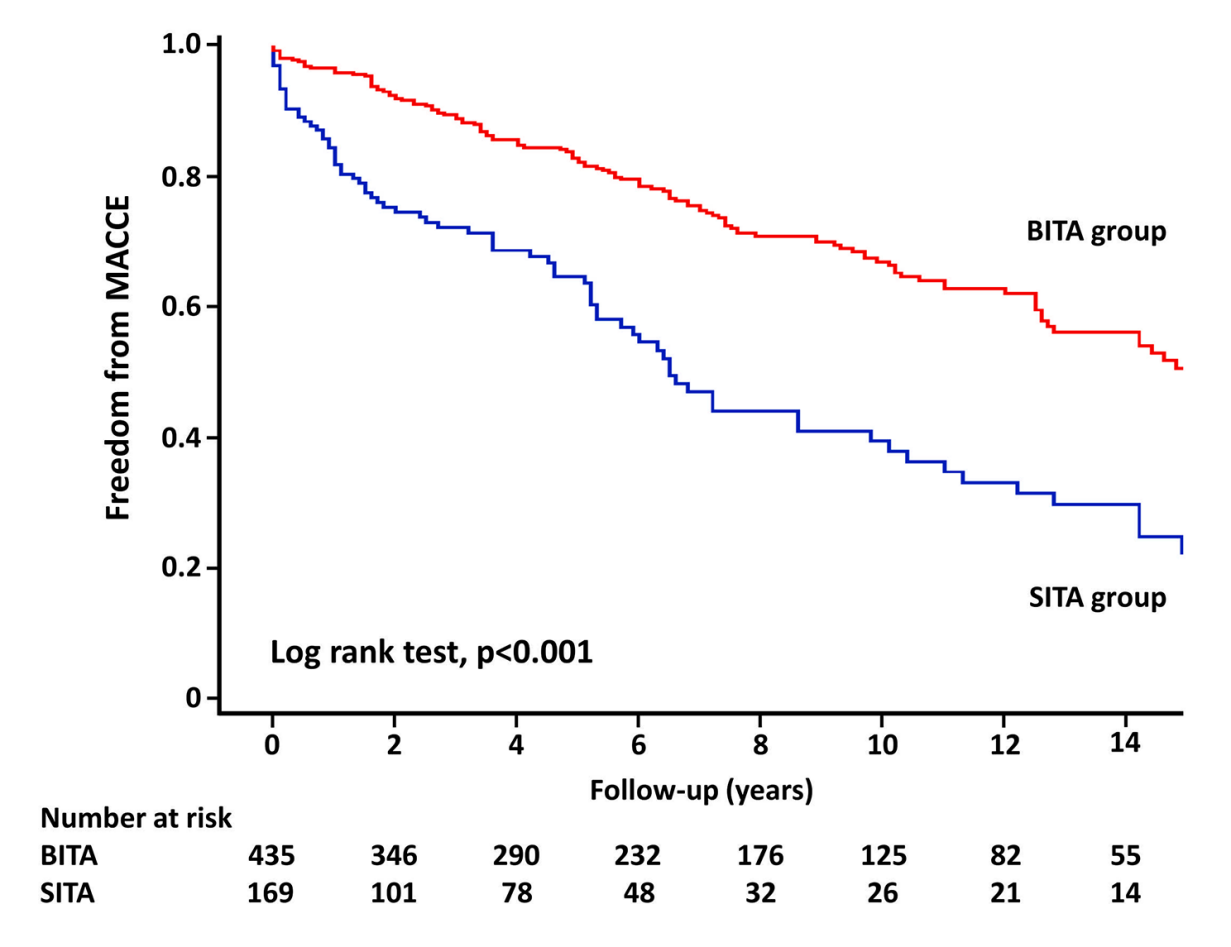
Freedom from MACCE in unweighted cohort

**Fig. 3:**
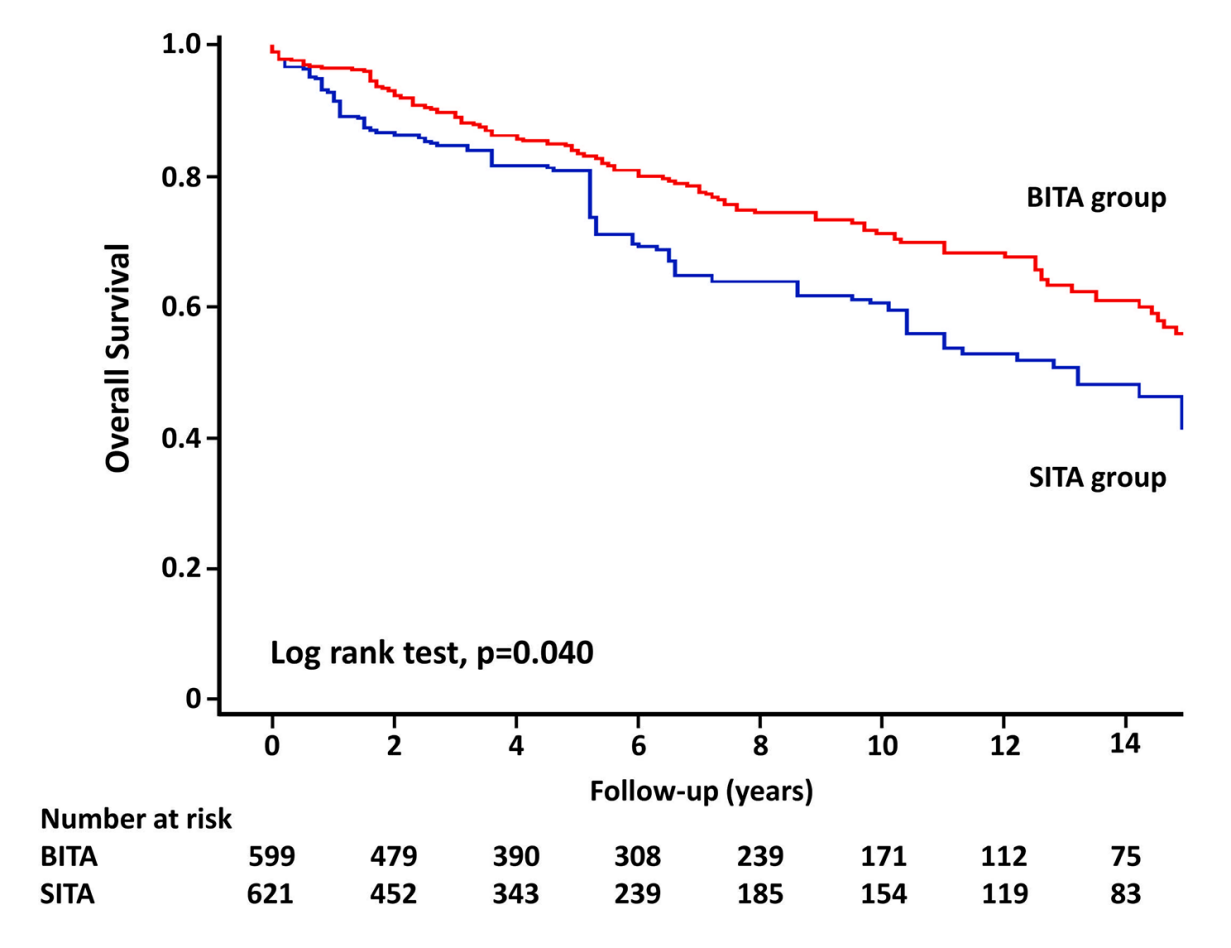
Overall survival in weighted cohort

**Fig. 4:**
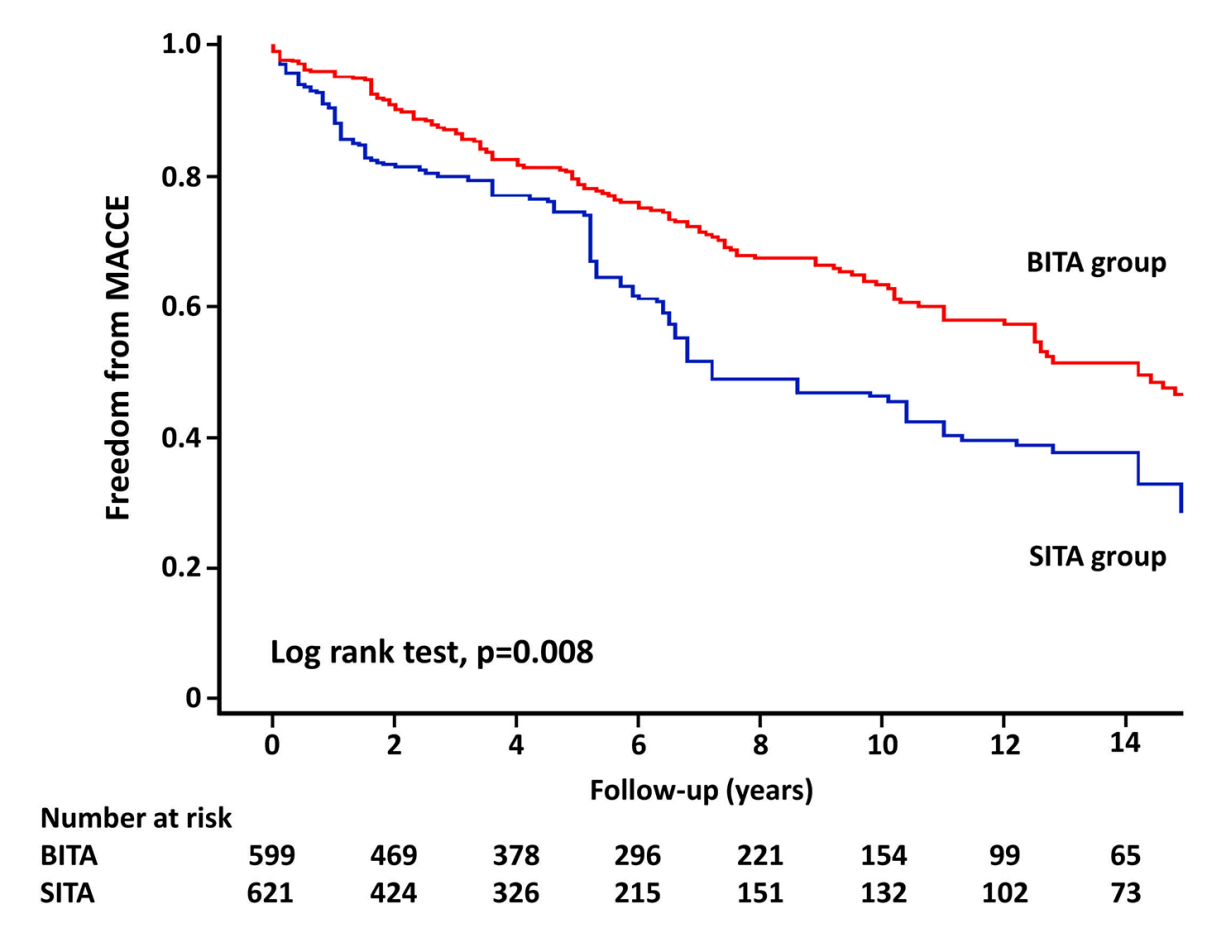
Freedom from MACCE in weighted cohort

Follow-up was completed in 98.7% (596/604) of the patients, and the mean follow-up duration was 6.7 years. All-cause death data, which includes patients who died within 30 days, are shown in Table 3. In unweighted cohort, the 10-year estimated rates of overall survival and freedom from MACCE, respectively, in the BITA group compared with the SITA group were 74.9% vs. 51.1% (Figs. 1) and 66.9% vs. 39.5% (Fig. 2); curves presented significant differences in all-cause death (*p* < 0.001) and MACCE (*p* < 0.001). In weighted cohort, the adjusted 10-year estimated rates of overall survival and freedom from MACCE, respectively, in the BITA group compared with the SITA group were 71.2% vs. 60.6% (Figs. 3) and 63.3% vs. 46.3% (Fig. 4); curves presented significant differences in all-cause death (*p* = 0.040) and MACCE (*p* = 0.008).

**Table 3:** Causes of all-cause death

	Unweighted			Weighted		
	BITA	SITA		BITA	SITA	
	(*n* = 435)	(*n* = 169)	*P* value	(SoW = 599.72)	(SoW = 621.25)	*P* value
All-cause death	104 (23.9%)	66 (39.1%)	< 0.001	159.09 (26.5%)	216.86 (34.9%)	0.002
Cardiac death	16 (3.7%)	15 (8.9%)	0.030	25.09 (4.2%)	46.12 (7.4%)	0.015
Myocardial infarction	1 (0.2%)	8 (4.7%)	0.007	1.30 (0.2%)	15.49 (2.5%)	0.001
Heart failure	12 (2.8%)	7 (4.1%)	0.383	19.87 (3.3%)	30.63 (4.9%)	0.155
Lethal arrhythmia	3 (0.7%)	0 (0%)	0.083	3.92 (0.7%)	0 (0%)	0.048
Noncardiac death	88 (20.2%)	51 (30.2%)	0.014	134.00 (22.3%)	170.74 (27.5%)	0.038
Pneumonia	18 (4.1%)	15 (8.9%)	0.049	25.36 (4.2%)	36.98 (6.0%)	0.171
Stroke	4 (0.9%)	3 (1.8%)	0.379	6.89 (1.1%)	9.20 (1.5%)	0.612
Sepsis	5 (1.1%)	6 (3.6%)	0.115	8.20 (1.4%)	14.69 (2.4%)	0.198
Cancer	18 (4.1%)	10 (5.9%)	0.351	25.74 (4.3%)	47.47 (7.6%)	0.013
Others	43 (9.9%)	17 (10.1%)	0.949	67.81 (11.3%)	62.40 (10.0%)	0.473

BITA: bilateral internal thoracic artery; SITA: single internal thoracic artery; SoW: sum of weights

Multivariate Cox proportional hazards analysis showed that BITA use was the predictor of all-cause death (hazard ratio [HR]: 0.706, 95% CI: 0.504–0.987; *p* = 0.042) and MACCE (HR: 0.671, 95% CI: 0.499–0.902; *p* = 0.008) (Table 4).

**Table 4:** Multivariate cox proportional hazards model for the predictors of all-cause death and MACCE

Predictor	HR	95% CI	*P* value
All-cause Death			
Age (year)	1.057	1.036–1.078	< 0.001
Body mass index (kg/m^2^)	0.992	0.944–1.043	0.755
Diabetes mellitus	2.102	1.513–2.919	< 0.001
Dyslipidemia	0.788	0.572–1.085	0.144
PAD	1.814	1.096–3.001	0.020
eGFR < 30 ml/min/1.73m^2^	3.786	2.619–5.472	< 0.001
Emergency operation	1.211	0.871–1.683	0.256
LVEF < 50%	1.579	1.142–2.183	0.006
BITA use	0.706	0.504–0.987	0.042
MACCE			
Age (year)	1.051	1.033–1.069	< 0.001
Body mass index (kg/m^2^)	0.990	0.947–1.035	0.647
Diabetes mellitus	1.636	1.238–2.163	0.001
Previous CVD	1.194	0.811–1.758	0.368
PAD	1.739	1.096–2.760	0.019
Thtree-vessel disease	1.360	0.971–1.905	0.074
eGFR < 30 ml/min/1.73m^2^	3.337	2.375–4.687	< 0.001
Emergency operation	1.283	0.962–1.710	0.090
LVEF < 50%	1.206	0.903–1.611	0.205
BITA use	0.671	0.499–0.902	0.008

BITA: bilateral internal thoracic artery; CI: confidence interval; CVD: cerebrovascular disease; eGFR: estimated glomerular filtration rate; HR: hazard ratio; LVEF: left ventricular ejection fraction; MACCE: major adverse cardiac and cerebrovascular events; PAD: peripheral artery disease

As subgroup analyses in our unweighted cohort, we compared late outcomes in (i) men vs. women, (ii) DM vs. non-DM and (iii) patients with LVEF < 50% vs. patients with LVEF ≧ 50%. The 10-year estimated rates of overall survival and freedom from MACCE, respectively, in men compared with women were 69.6% vs. 66.3% and 60.8% vs. 57.9% (Figure E4); curves presented no significant differences in all-cause death (*p* = 0.259) and MACCE (*p* = 0.251). The 10-year estimated rates of overall survival and freedom from MACCE, respectively, in DM compared with non-DM were 61.7% vs. 77.5% and 53.2% vs. 68.2% (Figure E5); curves presented significant differences in all-cause death (*p* < 0.001) and MACCE (*p* < 0.001). The 10-year estimated rates of overall survival and freedom from MACCE, respectively, in patients with LVEF < 50% compared with patients with LVEF ≧ 50% were 75.9% vs. 77.5% and 51.2% vs. 64.7% (Figure E6); curves presented significant differences in all-cause death (*p* < 0.001) and MACCE (*p* = 0.001).

## Discussion

BITA grafting has been reported to provide better long-term outcomes than SITA grafting in several diseases [6–9]. However, most such studies include a mixture of patients undergoing on-pump surgery and off-pump surgery. In the present study, we compared postoperative outcomes more precisely in patients with LMCAD after we reduced the influence of procedural confounding factors by including only patients who underwent off-pump surgery. To the best of our knowledge, the present report is the first one which reported the efficacy of BITA grafting compared to SITA grafting in patients with LMCAD who underwent isolated off-pump CABG.

A major finding of the present study was that the overall survival rate was significantly higher in the BITA than SITA group; BITA grafting was significantly associated with a lower risk for all-cause death after adjustment for potential confounders. One feasible explanation for this result is that ITA is more likely to be patent than the saphenous vein when grafted to the left coronary area at all times after surgery [12]. Additionally, in the PREVENT IV multicenter randomized trial, in which 3014 patients undergoing isolated CABG were enrolled, the incidence of vein graft failure at 12 months was significantly higher than that of ITA graft failure (25% vs. 8%) [13]. The ROOBY (Randomized On/Off Bypass) trial similarly showed that a vein graft failed more frequently that an ITA graft early after surgery, with or without cardiopulmonary bypass support [14]. We believe that off-pump BITA grafting provides better survival benefit in patient with LMCAD than SITA grafting.

Another major finding of the present study was that the rate of freedom from MACCE was significantly higher in the BITA than SITA group. Iribarne et al. investigated postoperative outcomes in 1297 propensity score-matched patients undergoing BITA or SITA grafting and showed BITA grafting was associated with a reduced risk of repeat revascularization than SITA grafting [15]. Barili et al. investigated postoperative outcomes in 10,988 patients who underwent isolated CABG in two large prospective multicenter cohort studies [16]. They concluded that BITA grafting was associated with a lower rate of repeat revascularization than SITA grafting after adjusting patients’ baseline characteristics using IPTW. Therefore, in addition to the survival benefit, the effectiveness of BITA grafting on postoperative lower rates of repeat revascularization may have affected the rate of MACCE at long-term follow-up in the present study.

The BITA group used more GEA and less saphenous vein than the SITA group (Table 2). When the right coronary artery system needed revascularization, the BITA group needed at least one graft in addition to BITA, because the BITA were anastomosed to the left coronary artery system. We aggressively used GEA as a third conduit in the BITA group when posterior descending artery had > 90% stenosis. Conversely, the GEA was used less in the SITA group because a saphenous vein was often used as a sequential graft to revascularize both the circumflex branches and the posterior descending artery. Previous studies showed the survival benefits of GEA grafting to the right coronary artery area [17, 18]. Additionally, we previously reported that the cumulative patency rate of in situ skeletonized GEA was 90.2% at 8 years after off-pump CABG [19]. Therefore, the survival benefit and better long-term patency of GEA may have affected the all-cause death and MACCE in the BITA group.

There were 1.6% (10/641) of patients who underwent CABG under cardiopulmonary bypass support in this study period, which included 5 patients with preoperative acute myocardial infarction. From this result, it seems that off-pump surgery was safely performed for patients with LMCAD in our cohort. LMCAD has historically been considered to carry a higher operative risk in patients undergoing CABG than those without LMCAD [20, 21]. Generally, off-pump surgery is not preferred in patients with LMCAD because the displacement of the heart could cause torsion of the left main trunk and acute hemodynamic deterioration [22]. In our institution, we revascularize LAD using in situ ITA at first during CABG surgery. Revascularization of the LAD does not require much displacement of the heart compared to that of the left circumflex artery and right coronary artery, so it is possible to perform anastomosis with less distortion of the left main trunk. Anastomosis to the left circumflex artery or right coronary artery area is done following confirmation of good bypass blood flow to the LAD during surgery. Therefore, the LAD is protected during anastomosis to the left circumflex artery or right coronary artery which requires strong displacement of the heart. This is probably the reason why we were able to complete off-pump CABG even in patients with LMCAD.

Previous studies report that BITA grafting during CABG increases the risk of postoperative deep sternal wound infection [23, 24]. In our cohort, there was no significant difference in postoperative deep sternal wound infection between the two groups (*p* = 0.227) (Table 2). Using the skeletonization technique when harvesting BTA grafts has been reported to reduce wound infection rates compared with pedicled harvesting [25, 26]. In the current study, all patients underwent CABG using the skeletonization technique, which may have contributed to this result.

### Study limitations

This study had several limitations. First, the study had a retrospective design with intrinsic selection bias. Despite statistical adjustments with IPTW, unmeasured confounders may have affected the postoperative outcomes. Second, all studied subjects were Japanese patients at a single center, which may limit generalizability. Finally, lack of available coronary angiographic data prevented evaluation of whether the survival benefit of BITA grafting is related to graft patency.

## Conclusions

Off-pump bilateral skeletonized ITA grafting is associated with lower rates of all-cause death and MACCE than SITA grafting in patients with LMCAD undergoing CABG without increasing postoperative complication.

### Electronic supplementary material

Below is the link to the electronic supplementary material.

Supplementary Material 1

Supplementary Material 2

Supplementary Material 3

Supplementary Material 4

Supplementary Material 5

Supplementary Material 6

Supplementary Material 7

## Data Availability

The data that support the findings of this study are available from the corresponding author, [K. Hachiro], upon reasonable request.

## Abbreviations

BITA: Bilateral internal thoracic artery
CABG: Coronary artery bypass grafting
CI: Confidence interval
DM: Diabetes mellitus
eGFR: Estimated glomerular filtration rate
GEA: Gastroepiploic artery
HR: Hazard ratio
IPTW: Inverse probability of treatment weighting
LAD: Left anterior descending artery
LMCAD: Left main coronary artery disease
MACCE: Major adverse cardiac and cerebrovascular events
PCI: Percutaneous coronary intervention
SITA: Single internal thoracic artery
SVG: Saphenous vein graft
