# Supplementary figures and images for: Off-pump bilateral internal thoracic artery grafting in patients with left main coronary artery disease
Source: J Cardiothorac Surg. 2024 Feb 9;19:81. doi: 10.1186/s13019-024-02582-5 (PMC10858637; doi:10.1186/s13019-024-02582-5)

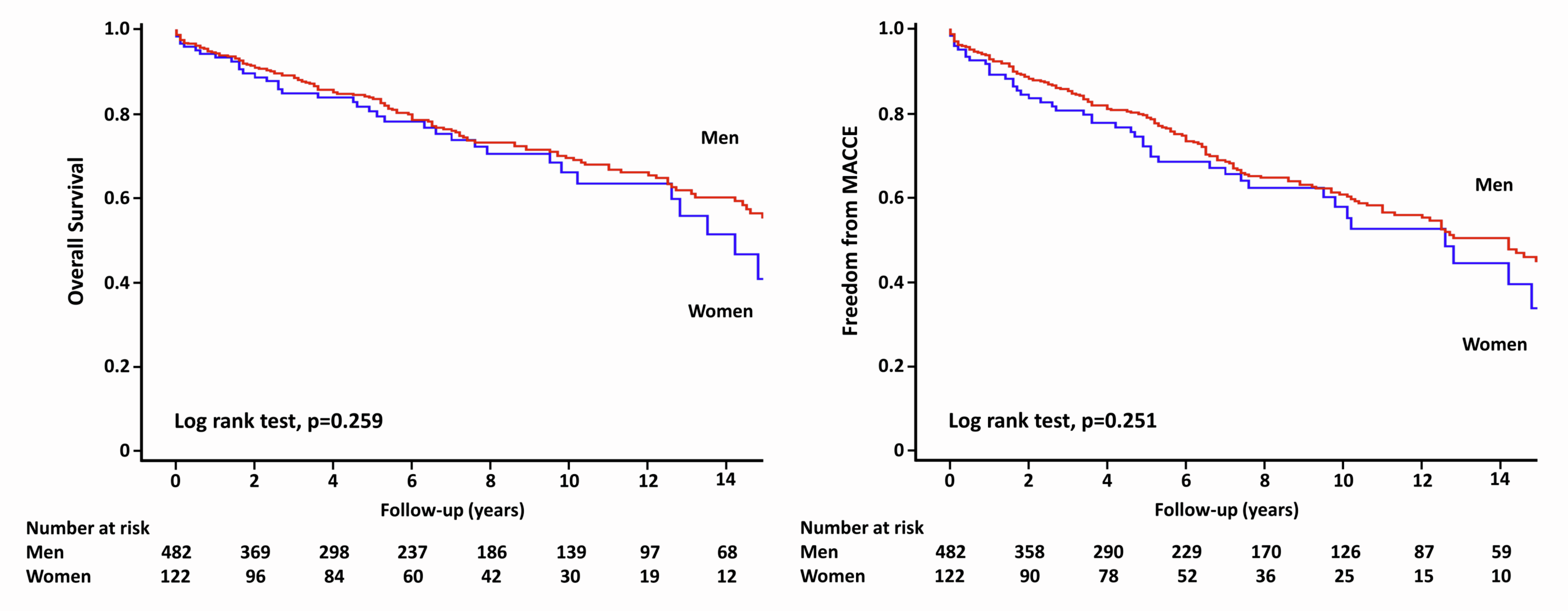

Supplement: Supplementary file 4 — Supplementary Material 4 [file 13019_2024_2582_MOESM4_ESM.tif]

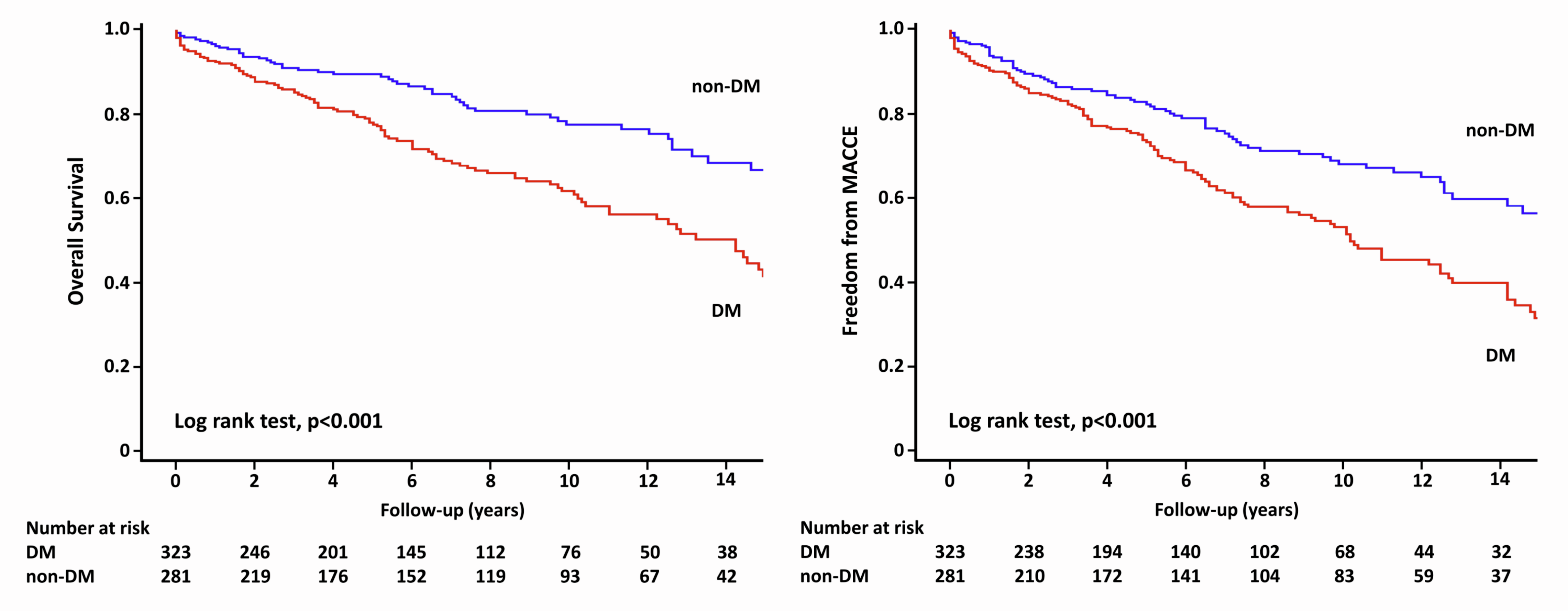

Supplement: Supplementary file 5 — Supplementary Material 5 [file 13019_2024_2582_MOESM5_ESM.tif]

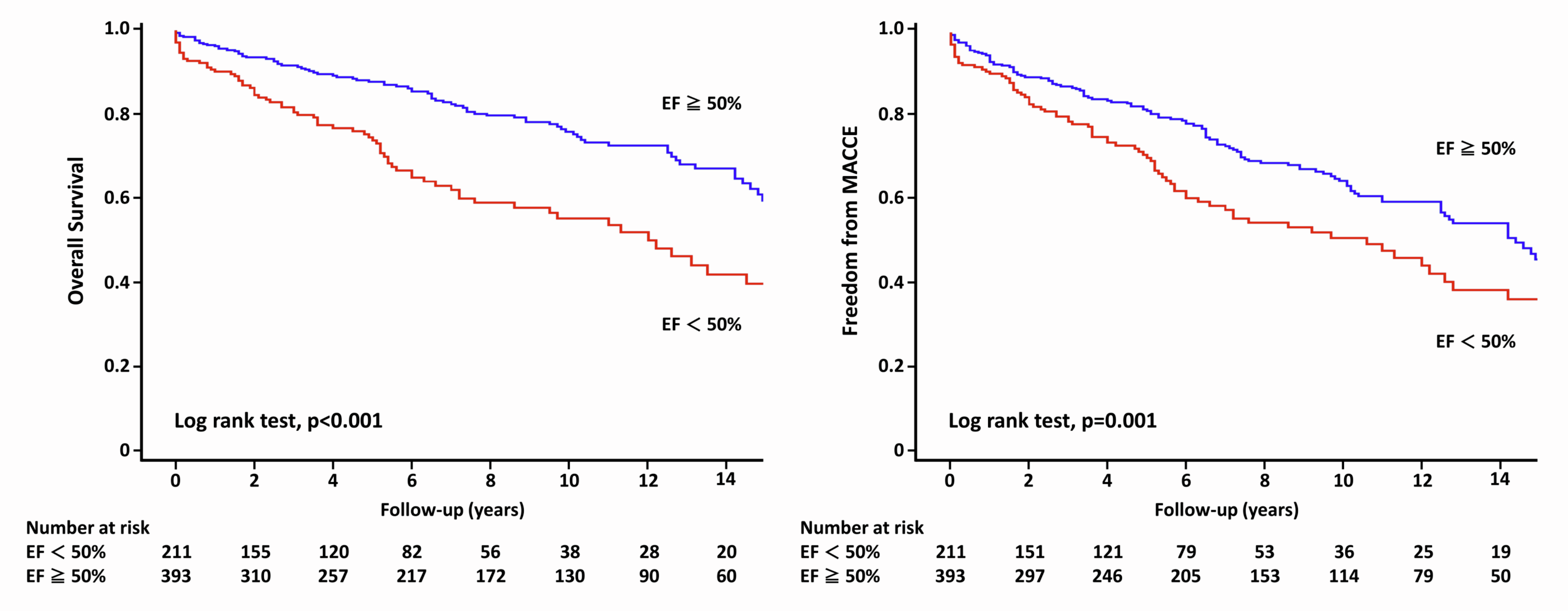

Supplement: Supplementary file 6 — Supplementary Material 6 [file 13019_2024_2582_MOESM6_ESM.tif]
