# Supplementary material for: Off-pump bilateral internal thoracic artery grafting in patients with left main coronary artery disease
Source: J Cardiothorac Surg. 2024 Feb 9;19:81. doi: 10.1186/s13019-024-02582-5 (PMC10858637; doi:10.1186/s13019-024-02582-5)
Supplement: Supplementary file 7 — Supplementary Material 7 [file 13019_2024_2582_MOESM7_ESM.docx]

Table E1. Univariate and Multivariate Logistic Regression Analyses for the Predictors of 30-day Mortality

| Table E1. Univariate and Multivariate Logistic Regression Analyses for the Predictors of 30-day Mortality | | | | | | |
| --- | --- | --- | --- | --- | --- | --- |
|  | Univariate | | | Multivariate | | |
|  | OR | 95% CI | P value | OR | 95% CI | P value |
| Age (year) | 1.045 | 0.974 - 1.122 | 0.221 |  |  |  |
| Sex (male) | 0.585 | 0.149 - 2.294 | 0.442 |  |  |  |
| Body mass index (kg/m^2^) | 0.867 | 0.705 - 1.066 | 0.176 |  |  |  |
| Hypertension | 0.928 | 0.237 - 3.630 | 0.914 |  |  |  |
| Diabetes mellitus | 3.543 | 0.746 - 16.824 | 0.112 |  |  |  |
| Dyslipidemia | 0.206 | 0.043 - 0.946 | 0.047 | 0.386 | 0.092 - 1.618 | 0.193 |
| Smoking history | 1.031 | 0.288 - 3.693 | 0.962 |  |  |  |
| Previous CVD | 0.832 | 0.104 - 6.664 | 0.862 |  |  |  |
| Previous PCI | 2.536 | 0.725 - 8.872 | 0.145 |  |  |  |
| PAD | 1.830 | 0.225 - 14.867 | 0.572 |  |  |  |
| Three-vessel disease | 1.257 | 0.264 - 5.986 | 0.774 |  |  |  |
| Hemoglobin A1c (%) | 0.502 | 0.206 - 1.225 | 0.130 |  |  |  |
| eGFR < 30ml/min/1.73m^2^ | 22.400 | 4.681 - 107.200 | < 0.001 | 22.856 | 5.538 - 94.327 | < 0.001 |
| Emergency operation | 4.174 | 1.068 - 16.308 | 0.040 | 2.987 | 0.651 - 13.695 | 0.159 |
| Acute MI | 1.407 | 0.392 - 5.043 | 0.600 |  |  |  |
| LVEF < 50% | 2.846 | 0.794 - 10.201 | 0.108 |  |  |  |
| Preoperative IABP | 4.604 | 1.309 - 16.186 | 0.017 | 2.213 | 0.567 - 8.644 | 0.253 |
| Operation time (m) | 1.003 | 0.993 - 1.013 | 0.533 |  |  |  |
| GEA use | 1.106 | 0.317 - 3.862 | 0.874 |  |  |  |
| SVG use | 1.560 | 0.447 - 5.449 | 0.486 |  |  |  |
| BITA use | 0.381 | 0.109 - 1.335 | 0.131 | 1.442 | 0.493 - 4.220 | 0.504 |
|  |  |  |  |  |  |  |

BITA: bilateral internal thoracic artery; CI: confidence interval; CVD: cerebrovascular disease; eGFR: estimated glomerular filtration rate; GEA: gastroepiploic artery; IABP: intra-aortic balloon pumping; LVEF: left ventricular ejection fraction; MI: myocardial infarction; OR: odds ratio; PAD: peripheral artery disease; PCI: percutaneous coronary intervention; SVG: saphenous vein graft
